# Expression of caspase-8 in non-alcoholic fatty liver disease related to ligature-induced periodontitis in rats

**DOI:** 10.1590/1678-7765-2025-0717

**Published:** 2026-03-30

**Authors:** Felipe Rodolfo Pereira da Silva, Victor Brito Dantas Martins, Hélio Mateus Silva Nascimento, Paulo Roberto Carneiro Gomes, Nikaely Brandão Barbosa, Vinícius da Silva Caetano, Ivana Márcia Alves Diniz, Karine Duarte da Silva, Any Carolina Cardoso Guimarães Vasconcelos, Daniel Fernando Pereira Vasconcelos

**Affiliations:** 1 Universidade Federal do Pará Faculdade de Medicina Altamira PA Brasil Universidade Federal do Pará (UFPA), Faculdade de Medicina, Altamira, PA, Brasil.; 2 Universidade Federal do Delta do Parnaíba Laboratório de Análises e Preparo Histológico Parnaíba PI Brasil Universidade Federal do Delta do Parnaíba (UFDPar), Laboratório de Análises e Preparo Histológico (LAPHis), Parnaíba, PI, Brasil.; 3 Universidade Federal de Minas Gerais Faculdade de Odontologia Departamento de Dentística Restauradora Belo Horizonte MG Brasil Universidade Federal de Minas Gerais (UFMG), Faculdade de Odontologia, Departamento de Dentística Restauradora, Belo Horizonte, MG, Brasil.; 4 Universidade Federal de Minas Gerais Faculdade de Odontologia Departamento de Patologia e Cirurgia Oral Belo Horizonte MG Brasil Universidade Federal de Minas Gerais (UFMG), Faculdade de Odontologia, Departamento de Patologia e Cirurgia Oral, Belo Horizonte, MG, Brasil.; 5 Instituto de Educação do Vale do Parnaíba Afya Faculdade de Medicina Parnaíba PI Brasil Instituto de Educação do Vale do Parnaíba (IESVAP - Afya), Faculdade de Medicina, Parnaíba, PI, Brasil.

**Keywords:** Apoptosis, Periodontal Disease, Liver dysfunction, Oxidative stress

## Abstract

**Aim:**

This study aimed to evaluate the oral and hepatic changes, as well as the expression of caspase-8 in liver tissue damage, both associated with ligature-induced periodontitis in rats.

**Methodology:**

A total of 16 female Wistar rats were divided into two groups (n=8): one received ligature around the first lower molar to promote periodontitis, and the other was the control group with no ligature. After the period of induction of the disease the animals were evaluated by clinical measures for periodontitis, euthanized, and the samples of gingival tissue, hepatic tissue, and serum were collected to the measurement of biomarkers for inflammation (myeloperoxidase), oxidative stress (glutathione, malonaldehyde, and nitrate [NO_3_]), liver damage and histopathological evaluation with immunohistochemistry for caspase-8. The data were expressed as mean and standard deviation for data with normal distribution or median and interquartile range for data with non-normal distribution. We used ANOVA followed by the Student-Newman-Keuls test for multiple comparisons of normally distributed data, and the Kruskal-Wallis test followed by Dunn's test for non-normally distributed data. The statistical tests were performed with GraphPad Prism Software (version 5.0), in which a p-value <0.05 was considered significant.

**Results:**

Our study demonstrates that the group with ligature-induced periodontitis showed increased measurement of periodontal destruction, local and systemic biomarkers of inflammation as well as liver damage which we observed several hepatocytes with loss of conformation and steatosis in the periodontitis group. The histopathological evaluation evidenced the periodontitis-related steatosis and higher expression of Caspase-8 in comparison with the control group (p<0.0006).

**Conclusion:**

Our study demonstrates the high expression of Caspase-8 in liver damage related to ligature-induced periodontitis in rats.

## Graphical abstract

**Figure:**
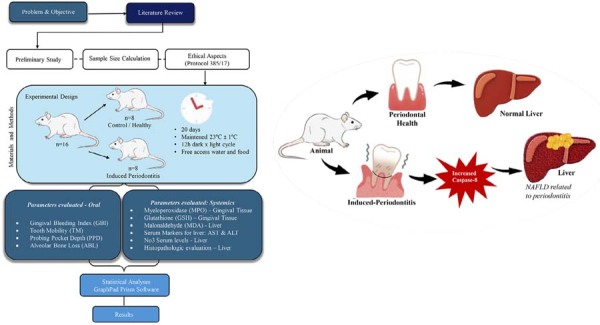

## Introduction

Periodontal diseases include a group of inflammatory diseases with multifactorial etiology influenced by environmental factors and genetic variations, in which the immune response of the host against oral pathogens results in destruction of the support tissues around teeth.^1^ The prevalence of these clinical findings is high, data show one billion cases of periodontal disease worldwide, with incidence in some countries such as Gambia and Denmark.^2,3^

The inflammatory process in periodontal diseases is complex, promoting changes not only locally but also resulting in systemic damage.^4^ Evidence shows the association among renal,^5^ and hepatic changes with periodontitis.^6,7^

One of the several liver alterations related to periodontitis is the non-alcoholic fatty liver disease (NAFLD), considered by the development of fatty infiltration in the hepatic cells in the absence of secondary causes, including the excessive alcohol consumption.^8^ NAFLD has considerable global prevalence reaching the value of 25.2% of population with the highest prevalence in Africa, Middle East, and South America.^9^

This association shows that lipopolysaccharides (LPS) from periodontopathogens act as inducers of liver changes.^10^ This hypothesis is reinforced by other studies that describe, in more detail, what is shown in liver samples from animals submitted to the ligature periodontitis model.^11-14^

The pathogenesis of NAFLD is well-hallmarked by the stimulation of pro-inflammatory cytokines in adipose and hepatic tissues. Oxidative stress biomarkers levels are altered in liver tissue because of inflammatory stimulus. As an example, previous data showed the influence of periodontitis in the levels of glutathione, malonaldehyde, and nitrate (NO_3_).^15,16^

Regarding the immune mediators with considerable role in NAFLD damage, the Tumor Necrosis Factor α (TNF-α) may be cited. TNF-α signal transduction is mediated by TNF-receptor 1 (TNFR1) that culminates with the formation of a protein complex and consequent activation of nuclear factor kB (NFkB) pathway and then the triggering of initiator aspartate‐specific cysteine protease (caspase-8).^17^

Caspase-8 is effective in the activation of the caspase system that cleaves cellular products and thereby induce the apoptotic cell death being closely related to liver cell damage.^18^ Besides, previous data showed increased levels of caspase-8 in the gingival crevicular fluid from individuals diagnosed with periodontitis.^19^

Seen these reports, this study aims to evaluate the oral and liver changes caused by periodontitis and verify the caspase-8 expression in liver tissue damage related to induced periodontitis in rats.

## Methodology

### Ethical aspects

The Ethics Committee of the Laboratory Animal Center of the Federal University of Piaui-UFPI (Protocol 385/17) approved all procedures in this research. A total of 16 female Wistar rats were maintained at 23°C ± 1°C, relative humidity of 67% ± 7% under a 12h dark x light cycle. The animals had free access to water and food, *Ad libitum* ration, composed of a base of cereals, vegetable protein, vitamins, and minerals. They were under these conditions for seven days prior to the procedures for acclimation. The study was conducted in accordance with ARRIVE guidelines.

### Sample size calculation

A priori sample size calculation was conducted to ensure adequate statistical power to detect biologically relevant differences between groups. Sample size estimation was performed using G*Power 3.1 for a one-way ANOVA design (fixed effects, omnibus test). The analysis assumed a two-sided alpha level of 0.05, a statistical power (1 − β) of 0.95, and an effect size of f = 0.6 (corresponding to a large effect according to Cohen’s conventions), based on evidence from studies in ligature-induced periodontitis models,^18,19^ as well as pilot data obtained by our group using identical procedures and outcome measures. Under these parameters, the calculation indicated that n = 8 animals per group would provide sufficient power (95%) to detect the expected differences.

### Experimental design

The animals were randomly divided into two groups (n=8 in each group) as follows: the control group composed of healthy rats, and the periodontitis group composed of animals that received a ligature for periodontitis inducing. The anesthesia was performed by intramuscular injection of a solution of 17 mg/Kg of 2.5% xylazine hydrochloride (Rompum-Bayer^®^, São Paulo, SP, Brazil) and 41 mg/Kg of ketamine (Francotar-Virbac^®^, Roseira, SP, Brazil). A nylon ligature (3-0, Shalon^®^, Goiania, GO, Brazil) was inserted around the periodontal region of the first mandibular molar of each animal.^20^ Blood was collected for biochemical investigations and after 20 days the animals were euthanized. Then, body and livers weights were measured and labelled in absolute (g) and relative (percentage, organ weight x 100/entire animal weight) values.

### Gingival bleeding index (GBI) evaluation

The measure of the periodontal pockets and/or gingival sulcus of the 1^st^ lower molar has been performed by the insertion of a probing for ten seconds and graded into the scores of 0, 1, 2, 3, 4, and 5.^21^ For the evaluation of clinical parameters, we used a round-tipped probe adapted with a tip radius of 0.4 mm, associated with artificial light and a high-resolution camera.

### Tooth mobility (TM) evaluation

TM was assessed in the first lower molar with a periodontal probe in the labiolingual direction (lateral plane) with the following scores: 0, physiological mobility; 1, slight mobility; 2, moderate mobility; 3, pronounced mobility.^21^

### Probing pocket depth (PPD) assessment

Mesiobuccal, distobuccal, and midbuccal points have been measured with the calculation of the mean as previously described.^6^ PPD values were evaluated using a round-ended probe (tip 2/10 mm in radius).

### Evaluation of alveolar boneloss (ABL)

The semi-mandibles were stained with a solution of methylene blue at 2%. The assessment of ABL was executed according to Vasconcelos, et al.^22^ (2020). Measurements were obtained along the longitudinal axis of the root as follows: (a) ABL-1, defined as the distance from the cemento-enamel junction (CEJ) to the alveolar crest at the anterior (mesial) aspect of the mandibular first molar; (b) ABL-2, determined by measuring the distance between the CEJ and the alveolar crest at the mesial root of the mandibular first molars; and (c) ABL-3, established by measuring the distance from the CEJ to the alveolar crest at the intermediate root of the mandibular first molars. And the images were obtained for histopathological assessment using an image examination system (ImageJ v.1.48 Software).

### Myeloperoxidase (MPO) activity

Neutrophil infiltrate was assessed in samples of gingival tissue from area with ligature-induced periodontitis. Concisely, 55 mg of tissue was homogenized at 55 mg/mL in potassium buffer containing 0.55% hexadecyltrimethylammomium bromide (HTAB). The homogenate was centrifuged in 4.000x g for 420 seconds at 5°C. The pellet was resuspended, and myeloperoxidase activity was assayed by measuring the change in absorbance at 450 nm, in ELISA reader, using o-dianisidinedihydrochloride and 1% hydrogen peroxide. Myeloperoxidase activity was described as units/mg of tissue. A unit of myeloperoxidase activity was defined as converting 1 μmol of hydrogen peroxide to water in 60 seconds at 23°C.^21^

### Glutathione assay (GSH)

The liver tissue was homogenized in 250 μl of a 5% gingival tissue solution prepared from 0.02M ethylenediaminetetraacetic acid (EDTA), in which 315 μL of distilled water and 88 μL TCA at 55% were added, the samples were centrifuged at 3,000 rpm for 0.4 hour at 4°C. Part of the supernatant (440 μL) was added to 880 μL of 0.4M Tris buffer at 8.8 (hydrogen potential - pH) with 25 μL of 0.01M 5,5′-Dithiobis (2-nitrobenzoic acid) (DNTB). Subsequently, the absorbance of each sample was evaluated at 412nm, and data were obtained with a GSH per mg of tissue.^20^

### Malonaldehyde assay (MDA)

Malonaldehyde levels were evaluated to determinate the lipid peroxidation in liver tissue. The solution was homogenized and 250 μl of 10% tissue was prepared in 1.16 KCL. Then, 3 ml of 0.55% thiobarbituric acid solution were added to 500 μl of the homogenate in a tube. This mixture was placed in a water bath for 46 min at 100°C, 4 milliliters of n-butanol were added, and the mixture was centrifuged. Absorbance was measured at 520 nm (A1) and 532 nm (A2), in which the amount of malondialdehyde was calculated as (A2-A1), performed in nmol MDA per gram of gingival tissue.^22^

### Serum markers for liver function evaluation

Liver function was assessed by measuring Alanine Aminotransferase (ALT), Aspartate Aminotransferase (AST), total cholesterol, triglycerides, and fractions serum levels with commercial kits (Labtest^®^, MG, Brazil).

### NO3 serum levels

NO_3_ serum levels were evaluated by the measurement of nitrite (NO_2_), the stable product of decomposition, using the Griess reaction and according Vasconcelos, et al.^14^ (2019). The following materials were used to freshly prepare Griess reagent each time before use for NO estimation: ortho-phosphoric acid (Merck KGsA, Damstadt, Germany), purified sodium nitrite (Merck KGsA, Damstadt, Germany), N-(1-naphthyl) ethylenediamine dihydrochloride (Merck KGsA, Damstadt, Germany), sulfanilamide (4-aminobenzene sulphonamide) (Bio Basic Inc., Markham, ON, Canada).

### Histopathologic evaluation of hepatic tissue

Hepatic samples were stored in formaldehyde solution after euthanasia and the histologic treating was performed^14^ for the left lobe; sections of 5 µm of thickness were obtained. The sections were stained with eosin and hematoxylin. The slides (15 sections per liver at 600 times original magnification) were observed on a light microscope. The histological evaluation of hepatic tissue has followed the parameters of: (a) hepatic steatosis, (b) inflammation, and (c) necrosis. The steatosis score was evaluated and classified according to the percentage of the cells with steatosis, within a scale of 5 degrees: 0, absent, or present in 4 outbreaks/field.^14^

### Immunocytochemistry for caspase-8

The eight slides per rat used to evaluate liver tissue were washed two times with phosphate buffered saline (PBS) containing 1.0 mM CaCl2 and 0.6 mM MgCl2. Cells fixed with 5% paraformaldehyde for at least three hours at 5°C. Coverslips containing fixed cells were washed three times with Tris buffered saline (TBS50 mM Tris-HCl, 0,14 M NaCl, 3%) pH 7.7 and then permeabilized with TBS containing 2% Bovine serum albumin (BSA) and 0.15% Triton X for 600 seconds. The first step was the inhibition of endogenous peroxidase to avoid its reaction with the substrate outside of the specific antigenic sites. For this, cells were incubated for 300 seconds with Peroxidase Block (NovocastraTM Peroxidase Detection System - RE7101), and then washed twice with TBS. The second step was the incubation for 300 seconds with a blocking protein (NovocastraTM Peroxidase Detection System - RE7101) to suppress non-specific binding of subsequent reagents. Subsequently cells were washed twice with TBS for 300 seconds.

For caspase-8 localization, a monoclonal mouse anti caspase-8 antibody (NovocastraTM Peroxidase Detection System - RE7101) was diluted 1:25 and applied for one hour at room temperature in a moist chamber. This antibody recognizes caspase-8 protein in its active form. Cells were washed with TBS and incubated with biotinylated secondary antibody (NovocastraTM Peroxidase Detection System - RE7101) for 30 minutes at room temperature. After incubating and washing the excess secondary antibody with TBS, cells were then incubated for 30 minutes with streptavidin-HRP (NovocastraTM Peroxidase Detection System - RE7101). Lastly, after washing cells with TBS, cells were stained with 3,3 ‘-diaminobenzidine in a buffer solution. Caspase-8 positive cells were identified by the presence of a brown color staining with sharp outlines and homogeneous in cytoplasm.

### Statistical analysis

Mean (± standard deviation) and/or mode, value that performs most often, (minimum and maximum) were used to express the data. The Shapiro-Wilk statistical test was performed to verify the distribution and normality of the data. The significant difference among the groups was analyzed using the variance test (ANOVA) and Student-Newman-Keuls test for data with normal-distribution and the Kruskal-Wallis test for data with no-normal distribution, followed by Dunn’s test for multiple comparisons. Kappa test was used to analyze the reproducibility of data. Statistical tests were performed on specific statistical software (Graphpad Prism Software Version 5.0), and *p-*value was considered significant when <0.05.

## Results

Measurement of the body and liver weights of the rats showed a slight variation for these parameters in the periodontitis and control groups (Table 1). However, non-significant differences were observed among the groups.

**Table 1:** Results on the total body and liver weights (in grams).

Parameters assessed	Groups		
Weight	Control	Periodontitis	
**Body**	220.8	225.4	*p*>0.10
**Liver**	6.801	6.757	*p*>0.10

### Clinical parameters of periodontal inflammation

**Figure 1:**
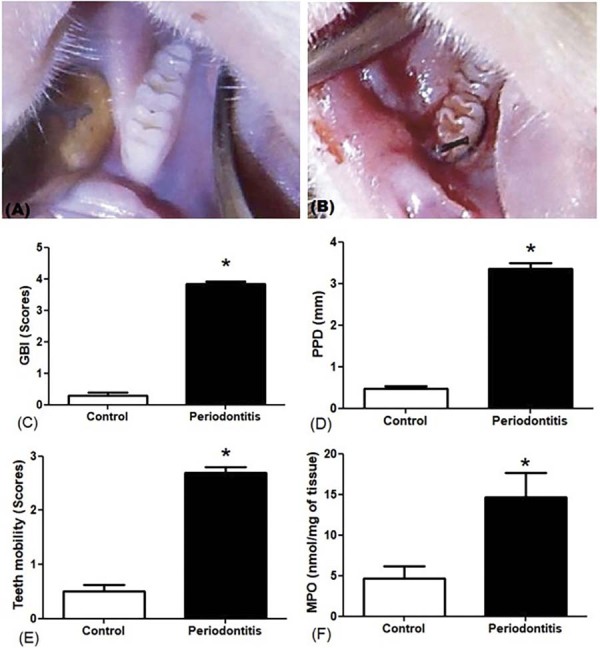
The image shows that the lower molar of the control group presents healthy periodontium (A). The group of periodontitis of the lower molars with the presence of ligation associated with edema and bacterial plaque around the lower first molar (B); The values of GBI, PPD, Tooth Mobility, and Gingival MPO levels were measured and presented a statistically significant difference between the periodontitis and the control group (C, D, E, F, respectively).

Figures 1A and 1B showed that the periodontitis was successfully induced by the ligature, and the mean of GBI, tooth mobility, and PPD were increased in the periodontitis group when compared with the control group (Figures 1C, 1D, and 1E) (GBI = 3.83±0.38, *p*<0.0001; Tooth mobility = 2.68±0.47, *p*<0.0001; PPD = 3.35±0.67, *p*=0.0002).

### MPO levels

The neutrophil infiltrate was estimated by the gingival MPO levels. The results demonstrated that animals with periodontitis have higher levels of MPO in comparison with the control group (Figure 1E) (Periodontitis = 14.65±9.372, Control = 4.771±4.681; *p*<0.0077).

### ABL measuring

ABL values were significantly higher in the periodontitis group when compared with the control group (Periodontitis = 5.32±0.58; Control = 1.99±0.23; *p*<0.05).

### GSH, MDA, and NO3 measurements

**Figure 2:**
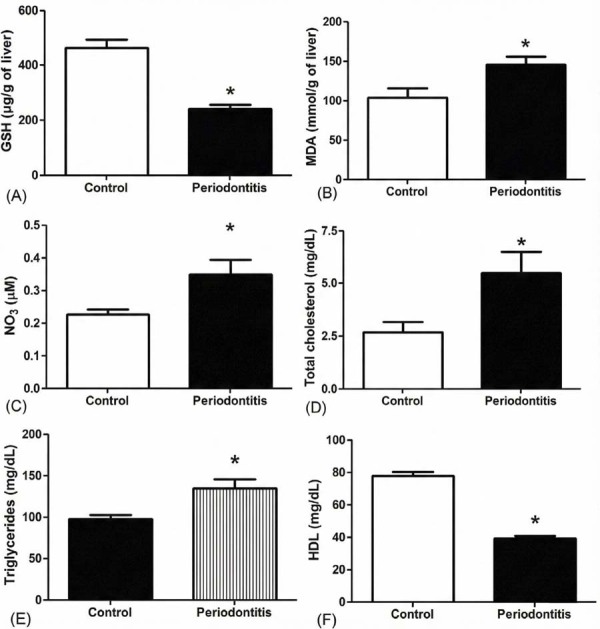
The images A and B show the means and standard deviation of the GSH and MDA measures of hepatic tissue, respectively; the images C, D, E, and F bring the data for nitrate, cholesterol, triglicerydes, and HDL serum levels between the periodontitis and control groups.

We observed a decreased value of GSH levels in the periodontitis groups (Periodontitis = 241.7±44.14 μ/g of tissue, Control = 463.8±104.8 μ/g of tissue; *p*<0.0001). Figure 2A shows that the group under ligature-induced periodontitis had about 48% less GSH levels than the control group. Lipid peroxidation in hepatic tissue from periodontitis group was higher compared to the control group by means of MDA levels measuring (Figure 2B) (Periodontitis = 145.3±34.05, Control = 103.3±42.47; *p*<0.018). Moreover, NO_3_ levels were higher in the periodontitis group in comparison with control group (Figure 2C) (Periodontitis = 0.3502±0.139, Control = 0.2272±0.04419; *p*<0.0015).

### Triglyceride, total cholesterol, and fractions results

The lipid profile was measured in the liver and serum of the animals by means of the triglycerides, cholesterol total, and fractions (Figures 2D, 2E, and 2F). There was an increase of about 44% in the levels of triglycerides in the hepatic tissues of the rats with periodontitis when compared to the control group (Periodontitis = 134.3±23.75, Control = 97.24±13.33; *p*<0.01). Similar results were found for values of triglycerides from the serum (Periodontitis = 99.03±33.53, Control = 71.25±19.38; *p*<0.0274). Cholesterol levels were higher in rats with periodontitis when compared with control (Periodontitis = 5.50±3.04, Control = 2.70±1.39; *p*<0.0233). HDL was measured in whole blood, whereas there was a significant increase in HDL in the control group when compared to animals with periodontitis (Periodontitis = 39.17±5.65, Control = 77.75±9.75; *p*<0.0001).

### ALT and AST results

The results suggest the association between periodontitis and liver alterations, since there was a higher production of both enzymes, ALT (Periodontitis = 33.80±6.28, Control = 28.53±8.67; P<0.132) and AST (Periodontitis = 95.09±18.32, Control = 82.17±20.54; *p*<0.1587), in the liver of animals with periodontitis.

### Histopathological evaluation of the liver

**Figure 3:**
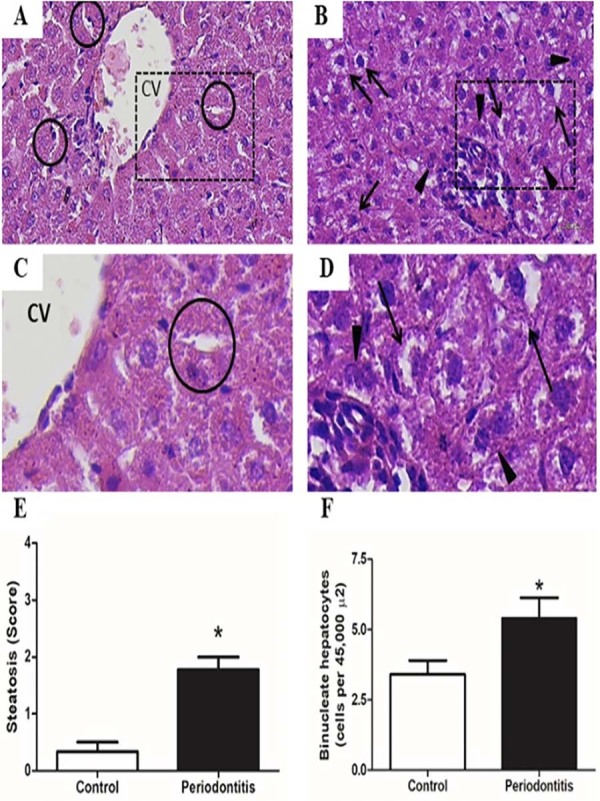
The image shows the hepatic tissue of the control group without histological changes, hepatocytes in normal conformation (A and B). C and D - represent the hepatic tissue of the periodontitis group demonstrating several hepatocytes with loss of conformation, showing steatosis. E and F bring data for the score of steatosis and measure of binucleated hepatocytes showing significant increased value for the periodontitis group.

**Figure 4:**
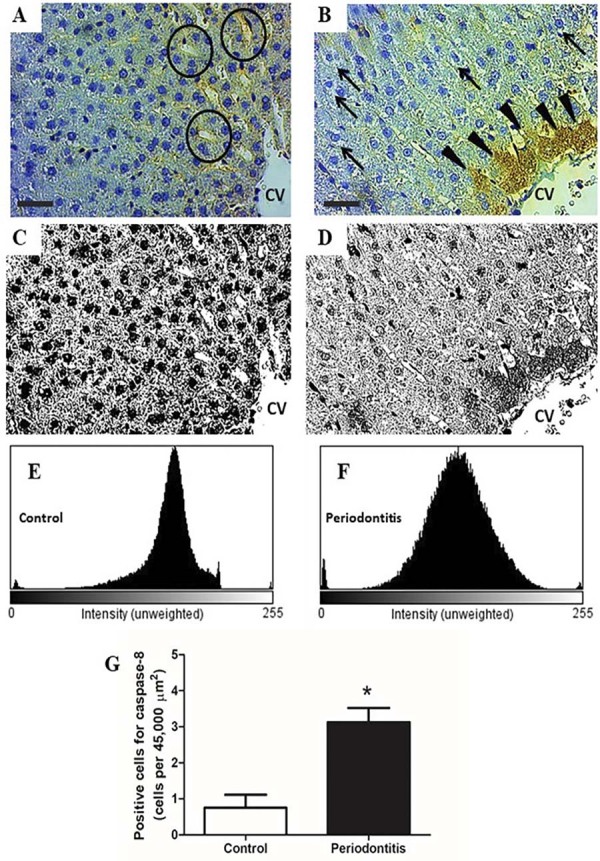
Photographs and quantification of positive cells for the caspase-8 (histogram and immunohistochemistry evaluation) from the groups. A illustrates normal liver from the control group, with the central vein (CV) visible in center of the image and the presence of sinusoid vessels (circles). B represents the hepatic tissue from the periodontitis group with several hepatocytes with microvesicular steatosis (arrows), hepatic cord rupture, with change in sinusoid trajectory and positive cells for caspase-8 (arrowheads). C and D show the same images from A and B, respectively, using the filter to highlight the difference in black and white. E reveals histogram evaluation of positive cells for the caspase-8 in the control group and F, the periodontitis group. G indicates the number of positive cells for the caspase-8, in which the periodontitis group has higher (p<0.05) than the control group. All photomicrographs are at 600× magnification. A and B immunohistochemistry for caspase-8. *p<0.05 vs control group.

There was a higher number of binuclear hepatocytes in animals with periodontitis when compared to the control group, respectively (Periodontitis = 5.40±2.32, Control = 3.40±1.57, *p*<0.0368) (Figures 3A, 3B, 3C and 3D). Moreover, we observed several hepatocytes with loss of conformation and steatosis in the periodontitis group (Figures 4B and 4D).

### Caspase-8 positive cell

Regarding caspase-8 expression in the liver tissue (Figure 4), we observed an increased number of positive cells for Caspase-8 in the rats with periodontitis when compared with the control group (Figures 4E, 4F and 4G) (Periodontitis = 3.12±1.13 vs Control = 0.75±1.03, *p*<0.0006).

## Discussion

To the best of our knowledge, this is the first study that assessed the caspase-8 expression in the liver alterations related to ligature-periodontitis rat model. The data demonstrated success in the induction of periodontitis by means of the parameters evaluated, according to the methodology proposed by Caetano et al.^20^(2022) and Vasconcelos, et al.^22^ (2017).

The periodontitis model by nylon ligature knotted around the periodontal region of the first mandibular molar of animals is commonly employed to mimic the periodontitis.^23,24^ The ligature around the teeth promote bacterial colonization, which leads to biofilm accumulation and development of symptoms of periodontitis as observed in a clinical situation.^25^ Data on GBI, TM, PPD, and ABL enabled the establishment of periodontitis and further performed analyses in our study.

To verify the inflammation on the periodontal sites, we measured the concentration of gingival MPO, which varies according to the inflammatory site. The MPO levels observed in the animals with periodontitis showed increased significant values when compared to the control group, which leads us to infer a high grade of inflammatory process in this group, as observed in previous data.^26^

When we consider the oxidative stress conducted by periodontitis, which is related with liver alterations, our data showed a decrease in GSH values and an increase in MDA levels in the hepatic samples from the ligature-induced periodontitis group. These results indicate the occurrence of oxidative stress triggering the consumption of antioxidants molecules such as GSH, which normally plays a protective role in the liver. This finding is corroborated by the increase in MDA levels, which indicates the oxidative stress status being found in high levels in periodontitis.^20^

Moreover, we verified other useful biomarker for oxidative stress evaluation: NO^3^. The assessing of NO^3^ levels in serum showed a significant increasing in the periodontitis group compared to the group without periodontitis, which is in accordance with previous findings.^26^ This molecule is loaded locally and may act as a cytotoxic factor against microorganisms but possibly results in tissue damage in the inflammatory process.

For liver evaluation, we measured biomarkers levels related to liver damage. Our results showed increased levels for AST and ALT in rats with periodontitis. These enzymes are commonly used to assess liver function that are present internally in hepatocytes. There is an increase in these enzyme levels when liver damage occurs^13^.

Besides, cholesterol and triglyceride levels were measured in the serum and liver samples from both groups, which showed significant higher levels in the periodontitis group. The increased levels in lipids profile have been previously related to diabetic animals, specifically rats with periodontal disease.^27^ Although the exact pathway involved in changes in the lipids profile and periodontitis remains unclear, the data indicated the role of periodontitis in systemic inflammation, dysmetabolic status, and dyslipidemia.

Histopathological assessment of liver tissue is required for liver NAFLD evaluation. In our experiment the analysis enabled the diagnosis of hepatic steatosis in rats with periodontitis, unlike what occurred in the liver tissue of the control group, which did not show histopathological alterations (Figure 4).

Our data showed increased Caspase-8 expression by quantifying the positive cells in the periodontitis group in comparison with the control group (Figure 4). As a protease with a dual role with pro-death and pro-survival functions mediating apoptosis by death receptors such as TNFR1 and suppressing necroptosis by pseudokinase MLKL and kinase RIPK3.^28^

We hypothesized the relation among caspase-8 expression, liver, and other tissue damage in periodontitis. Previous authors demonstrated the expression of caspase-3 in patients with periodontitis whose expression has increased as the progression of the disease.^29^ The increased caspase-8 mRNA expression was observed progressively with increasing fatty liver severity in rats with NAFLD.^30^ Furthermore, previous findings demonstrated the effectiveness in the treatment with specific drug for NAFLD by blocking caspase-8 signaling pathway that let reinforce the participation of this caspase in liver damage.^31^

The pathophysiological mechanism of apoptosis involves the intrinsic and extrinsic pathways with caspase activation.^32^ Extrinsic pathway initiates with the activation of cell death messengers to extracellular receptors such as Fas and TNFR1 with subsequent caspase-8 activation.^33^

In fact, the abnormal activation of TNF pathway leads to massive liver damage and the deletion of TNF receptor or caspase-8 expression results in the restoring of hepatic functions (17). TNF-α has stimulated, *in vivo*, the apoptosis of fibroblast via caspase-8 activation,^34^ with high immune-mediator in rats under the ligature-induced periodontitis and liver alterations.^14^ These findings led us to infer the participation of caspase-8 in the NAFLD related to periodontitis.

Our findings demonstrate an important contribution to understanding the damage caused by periodontitis, both in oral parameters and in the liver. However, other mechanisms in addition to these alterations can be further elucidated by various investigative approaches. Furthermore, different study models could provide more robust data on how liver alterations develop not only in ligature-induced periodontitis in animals, but also in clinical studies involving humans.

## Conclusion

In conclusion, we observed the increased expression of Caspase-8 in liver tissue from rats with ligature-induced periodontitis.

## References

[1] Silva FR, Pessoa LS, Shin JI, Alves EH, Koga RS, Smith CV (2021). Polymorphisms in the interleukin genes and chronic periodontitis: a field synopsis and revaluation by Bayesian approaches. Cytokine.

[2] Dai X, Dai M, Liang Y, Li X, Zhao W (2025). Global burden and trends of oral disorders among adolescent and young adult (10-24 years old) from 1990 to 2021. BMC Oral Health.

[3] Zhang X, Wang X, Wu J, Wang M, Hu B, Qu H (2024). The global burden of periodontal diseases in 204 countries and territories from 1990 to 2019. Oral Dis.

[4] Isola G, Polizzi A, Serra S, Boato M, Sculean A (2025). Relationship between periodontitis and systemic diseases: a bibliometric and visual study. Periodontol 2000.

[5] Galeno JG, França LF, Silva FR, Alves EH, Lenardo DD, Nascimento HM (2021). Renal alterations caused by ligature-induced periodontitis persist after ligature removal in rats. J Periodontal Res.

[6] Ayala KN, Caetano VD, Vasconcelos AC, Sousa MV, Barbosa NB, Mesquita LM (2025). Development of liver disease caused by chronic periodontitis in rats. J Appl Oral Sci.

[7] Zhang Z, Zheng Q, Liu Y, Chen G, Li Y (2025). Association between periodontitis and mortality in participants with metabolic dysfunction-associated steatotic liver disease: results from NHANES. BMC Oral Health.

[8] Leow WQ, Chan AW, Mendonza PG, Lo K, Yap K, Kim H (2023). Non-alcoholic fatty liver disease: the pathologist's perspective. Clin Mol Hepatol.

[9] Mitra S, De A, Chowdhury A (2020). Epidemiology of non-alcoholic and alcoholic fatty liver diseases. Transl Gastroenterol Hepatol.

[10] Pischke S, Shiprov A, Peters U, Schulze Zur Wiesch J, Kluwe J, Westphal T (2023). High prevalence of periodontal disease in patients with NASH-possible association of poor dental health with NASH severity. Ann Hepatol.

[11] Alves EH, Carvalos AS, Silva FR, França LF, Lenardo DD, Vasconcelos AC (2020). Bromelain reduces the non-alcoholic fatty liver disease and periodontal damages caused by ligature-induced periodontitis. Oral Dis.

[12] Andrade RS, França LF, Pessoa LS, Landim BA, Rodrigues AA, Alves EH (2019). High-fat diet aggravates the liver disease caused by periodontitis in rats. J Periodontol.

[13] Pessoa LS, Silva FR, Alves EH, França LF, Lenardo DD, Carvalho JS (2018). One or two ligatures inducing periodontitis are sufficient to cause fatty liver. Med Oral Patol Oral Cir Bucal.

[14] Vasconcelos AC, Vasconcelos DF, Silva FR, França LF, Alves EH, Lenardo DD (2019). Periodontitis causes abnormalities in the liver of rats. J Periodontol.

[15] Ferreira IL, Costa S, Moraes BJ, Costa A, Fokt O, Marinho D (2023). Mitochondrial and redox changes in periodontitis and type 2 diabetes human blood mononuclear cells. Antioxidants (Basel).

[16] Li Q, Ma H, Shang Y, Xin X, Liu X, Wu Z (2024). The role of uncoupling protein 2 in experimental periodontitis-associated renal injury in rats. Hua Xi Kou Qiang Yi Xue Za Zhi.

[17] Dara L (2018). The receptor interacting protein kinases in the liver. Semin Liver Dis.

[18] Gao H, Zhong Y, Zhou L, Lin S, Hou X, Ding Z (2023). Kindlin-2 inhibits TNF/NF-?B-Caspase 8 pathway in hepatocytes to maintain liver development and function. Elife.

[19] Aral K, Aral CA, Kapila Y (2019). The role of caspase-8, caspase-9, and apoptosis inducing factor in periodontal disease. J Periodontol.

[20] Caetano VS, Andrade RS, França LF, Pessoa LS, Rodrigues AA, Alves EH (2022). Food restriction reduces hepatic alterations associated with experimental periodontitis. J Periodontol.

[21] Vasconcelos AC, Vasconcelos DF, Silva FR, França LF, Alves EH, Lenardo DD (2020). Alpha-terpineol complexed with beta-cyclodextrin reduces damages caused by periodontitis in rats. J Periodontal Res.

[22] Vasconcelos DF, Silva FR, Pinto ME, Santana LA, Souza IG, Souza LK (2017). Decrease of pericytes is associated with liver disease caused by ligature-induced periodontitis in rats. J Periodontol.

[23] Molon RS, Mascarenhas VI, Avila ED, Finoti LS, Toffoli GB, Spolidorio DM (2016). Long-term evaluation of oral gavage with periodontopathogens or ligature induction of experimental periodontal disease in mice. Clin Oral Investig.

[24] Lee MJ, Ryu HH, Hwang JW, Kim JR, Cho ES, Choi JK (2023). Sirt6 activation ameliorates inflammatory bone loss in ligature-induced periodontitis in mice. Int J Mol Sci.

[25] Lin P, Niimi H, Ohsugi Y, Tsuchiya Y, Shimohira T, Komatsu K (2021). Application of ligature-induced periodontitis in mice to explore the molecular mechanism of periodontal disease. Int J Mol Sci.

[26] Ambati M, Rani KR, Reddy PV, Suryprasanna J, Dasari R, Gireddy H (2017). Evaluation of oxidative stress in chronic periodontitis patients following systemic antioxidant supplementation: a clinical and biochemical study. J Nat Sci Biol Med.

[27] Mirzaei A, Shahrestanaki E, Malmir H, Ejtahed HS, Tajbakhsh D, Seif E (2022). Association of periodontitis with lipid profile: an updated systematic review and meta-analysis. J Diabetes Metab Disord.

[28] Newton K, Wickliffe KE, Maltzman A, Dugger DL, Reja R, Zhang Y (2019). Activity of caspase-8 determines plasticity between cell death pathways. Nature.

[29] Pradeep AR, Suke DK, Prasad MV, Singh SP, Martande SS, Nagpal K (2016). Expression of key executioner of apoptosis caspase-3 in periodontal health and disease. J Investig Clin Dent.

[30] Li CP, Li JH, He SY, Li P, Zhong XL (2014). Roles of Fas/Fasl, Bcl-2/Bax, and Caspase-8 in rat nonalcoholic fatty liver disease pathogenesis. Genet Mol Res.

[31] Zhang Y, Zhou G, Chen Z, Guan W, Zhang J, Bi M (2020). Si-Wu-Tang alleviates nonalcoholic fatty liver disease via blocking TLR4-JNK and Caspase-8-GSDMD signaling pathways. Evid Based Complement Alternat Med.

[32] Tummers B, Green DR (2017). Caspase-8: regulating life and death. Immunol Rev.

[33] Prasun P, Ginevic I, Oishi K (2021). Mitochondrial dysfunction in nonalcoholic fatty liver disease and alcohol related liver disease. Transl Gastroenterol Hepatol.

[34] Alikhani M, Alikhani Z, Raptis M, Graves DT (2004). TNF-alpha in vivo stimulates apoptosis in fibroblasts through caspase-8 activation and modulates the expression of pro-apoptotic genes. J Cell Physiol.

